# Metastatic Malignant Melanoma of the Small Intestines Diagnosed by Capsule Endoscopy

**DOI:** 10.4021/gr342w

**Published:** 2011-09-20

**Authors:** Hussain Issa, Abduljaleel M. Poovathumkadavil, Fadel Almousa, Ahmed H. Al-Salem

**Affiliations:** a Department of internal medicine, Division of gastroentrology, Dammam, Saudi Arabia; b Department of Radiology, King Fahad Specialist Hospital, Dammam, Saudi Arabia; c Department of Pediatric Surgery, Maternity and Children Hospital, Dammam, Saudi Arabia

**Keywords:** Melanoma, Metastasis to intestines, Capsule endoscopy

## Abstract

Malignant melanoma is a fairly common tumor that shows an unusual predilection to metastasize to the small intestines. The time interval between the diagnosis of metastasizing melanoma and the initial diagnosis is variable. This as well as the non specific symptoms and the fact that the small bowel is inaccessible both radiologically and endoscopically lead to delay in diagnosis. We present a case of metastatic malignant melanoma to the small intestines diagnosed several years post excision by capsule endoscopy. Metastatic melanoma in the small bowel should be suspected in any patient with a previous history of malignant melanoma who develops non specific gastrointestinal symptoms. Capsule endoscopy which is non invasive, convenient to the patient and devoid of radiation should form part of their diagnostic investigation.

## Introduction

Melanoma is a relatively common malignant neoplasm that accounts for 1-3% of all cancers and has an unusual tendency to metastasize to the gastrointestinal tract [1-5]. The diagnosis of metastatic malignant melanoma to the gastrointestinal tract is usually difficult because the symptoms are none specific and the small intestines are not accessible to endoscopic investigations. The variable time interval between the diagnosis of primary malignant melanoma and the diagnosis of metastatic intestinal melanoma which may be prolonged makes the possibility of metastatic melanoma a remote possibility. This report describes an unusual case of metastatic malignant melanoma diagnosed by capsule endoscopy more than eight years post diagnosis.

## Case Report

**Figure 1:**
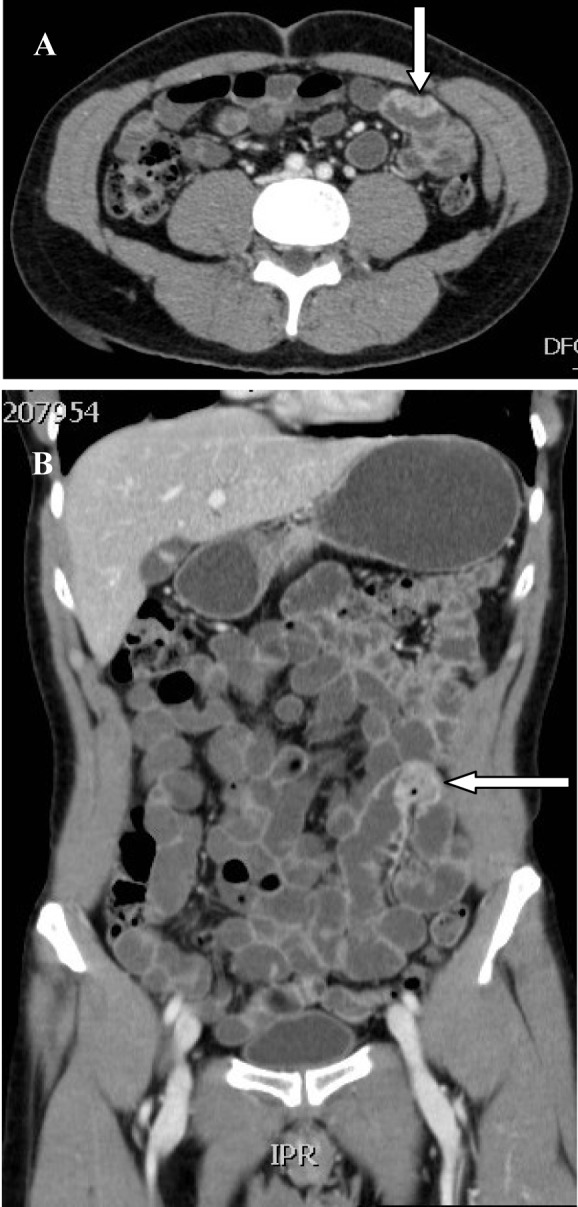
CT-scan showing metastatic tumour to the small intestines resembeling malignant melanoma in axial (A) and coronal sections (B).

**Figure 2:**
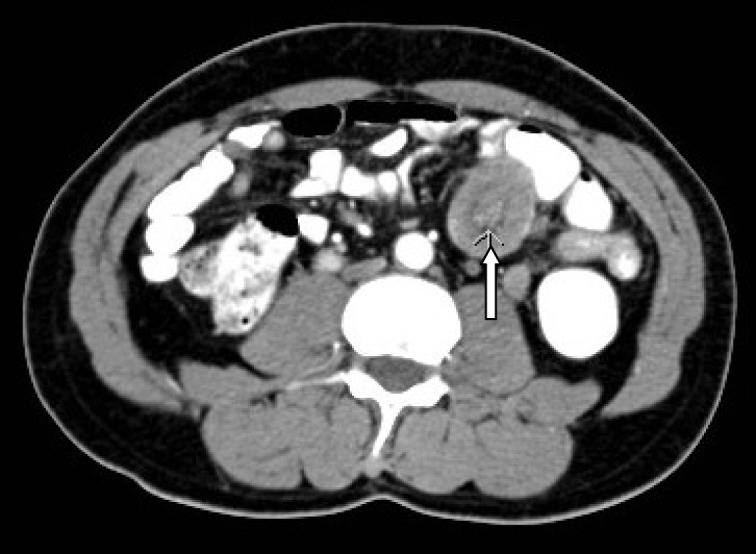
CT-scan showing small intestines intussusceptions secondary to a metastasizing malignant melanoma.

**Figure 3:**
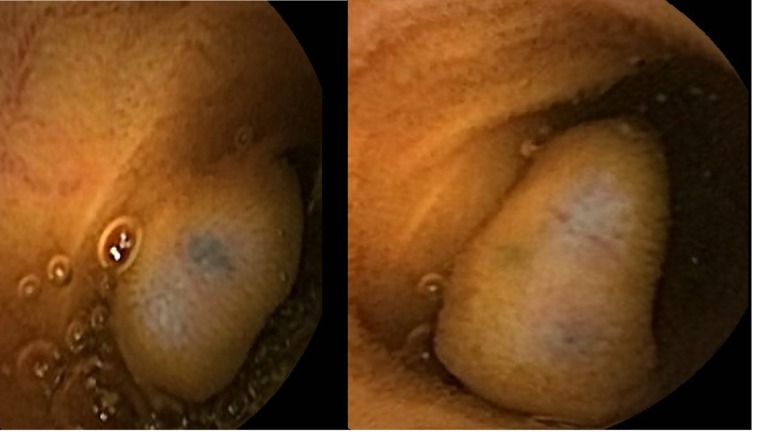
Clinical photograph from capsule endoscopy showing multiple areas of black nodules and in some areas black pointed polypoid mucosa of the jejunal loops.

A 54-year-old Saudi male, a known case of melanoma of the proximal phalanx of the right thumb presented to our hospital with non specific abdominal pain. He was diagnosed to have malignant melanoma and underwent amputation of the right thumb in 2002 for stage IIIB melanoma. Subsequently he received chemotherapy and follow up CT scan showed multiple pulmonary nodules. Fine needle aspiration confirmed a small cell carcinoma for which he received chemotherapy and radiotherapy. A repeat CT scan showed significant reduction of the size of the pulmonary nodules and eventually their disappearance leaving some fibrotic changes. He remained asymptomatic till September 2010 when a follow up PET scan showed focal discrete bowel wall activity in the proximal jejunal loops. CT scan of the abdomen showed mural nodular thickening of the proximal jejunal loops (Fig. 1) with intussusceptions but no signs of intestinal obstruction (Fig. 2). Capsule enteroscopy was performed and showed multiple areas of black nodules and in some areas black pointed polypoid mucosa of the jejunal loops (Fig. 3) indicative of metastatic melanoma. He remained asymptomatic, with a stable hemoglobin and negative occult bloodin the stools. Currently, he is atable and under close follow-up.

## Discussion

Primary malignant tumors of the small intestines are rare and the majority is adenocarcinoma and carcinoid tumor [2, 6]. Primary melanoma of the small intestines is extremely rare with less than 20 cases reported in the literature and because of this the diagnosis of primary malignant melanoma of the small intestines before it is made, primary malignant melanoma at a more common site should be excluded [1]. Metastatic tumors of the small intestines on the other hand are much more common than primary tumors. Among the various malignant tumors that may metastasize to the small bowel, malignant melanoma is considered the primary tumor in 50-70% of cases [1, 3, 4]. The small bowel is the most common site of involvement and of these the terminal ileum is the most commonly affected part followed by the stomach but colonic involvement is rare [7]. In one case series, melanoma commonly metastasized to the liver (68%), small intestine (58%), the colon (22%), and the stomach (20%) [8]. Malignant melanoma metastasizing to the gastrointestinal tract is usually a late manifestation of the disease with an overall poor prognosis [9]. Most often, gastrointestinal metastatic melanoma is asymptomatic and is only discovered at post-mortem examination or present with non-specific symptoms like abdominal pain, nausea, vomiting, weight loss, GI bleeding and anemia [9-12]. These secondaries are slow growing and usually grow extraluminally. This makes it difficult to establish the diagnosis as the symptoms are usually non specific and the small intestines are not easily accessible endoscopically. The time interval between the diagnosis of primary malignant melanoma and the diagnosis of metastatic intestinal melanoma is variable ranging from 2-180 months [13, 14]. In our patient, the diagnosis of metastatic malignant melanoma to the small intestines was made more than eight years post excision of primary malignant melanoma of the right thumb. This calls for a close and prolonged follow-up of these patients. The possibility of metastasizing malignant melanoma to the intestines must be kept in mind and they should be investigated for such a possibility even if they present very late with non specific symptoms such as vague abdominal pain. Rarely, however they may present acutely because of intestinal obstruction secondary to intussusception and in these patients the diagnosis of metastasizing malignant melanoma to the intestines is made intraoperatively [15, 16]. Our patient had an episode of asymptomatic intussusception that reduced spontaneously. The diagnosis of this was made by CT-scan of the abdomen which also revealed features of metastasizing malignant melanoma. Several reports have described the radiological features of metastatic tumors to the small bowel. In the past, the diagnosis of small bowel tumors whether primary or secondaries is usually delayed due to the poor available diagnostic techniques and difficulty of endoscopic access to the small bowel. This however is not the case nowadays where an array of new diagnostic techniques have been developed increasing the possibility of diagnosing these tumors at an earlier stage. Among these capsule endoscopy is the most valuable [17]. It is non-invasive, convenient to the patient, enables visualization of the entire small bowel and without radiation exposure. An important point is the fact that the amount of melanin in metastatic lesions is variable in the same patient [18]. The reason for this is not known. This was the case in our patient. This may also explain the fact that the majority of polypoid metastatic lesions are amelanotic [19]. This fact must be born in mind in patient with a history of previous malignant melanoma as these may resemble simple polyps both radiologically and endoscopically.

In conclusion, metastatic melanoma in the small intestines should be suspected in any patient with a previous history of malignant melanoma and present with non specific gastrointestinal symptoms, anemia, gastrointestinal bleeding intussusception or weight loss. This is even so if the presentation is delayed for several years. Capsule endoscopy should form part of their evaluation. It is a non-invasive, well tolerated by patients, allows visualization of the entire small bowel with high-quality images and devoid of radiation exposure.
